# Unexpected Infection Spikes in a Model of Respiratory Syncytial Virus Vaccination

**DOI:** 10.3390/vaccines5020012

**Published:** 2017-05-18

**Authors:** Robert J. Smith?, Alexandra B. Hogan, Geoffry N. Mercer

**Affiliations:** 1 Department of Mathematics and Faculty of Medicine, The University of Ottawa, 585 King Edward Ave, Ottawa, ON K1N 6N5, Canada; 2 Department of Infectious Disease Epidemiology, Imperial College London, St Mary’s Campus, London W2 1PG, UK and Research School of Population Health, The Australian National University, Canberra 2601, Australia; a.hogan@imperial.ac.uk; 3 Research School of Population Health, The Australian National University, Canberra 2601, Australia; veganaxos@gmail.com

**Keywords:** Respiratory Syncytial Virus, vaccination, mathematical model, impulsive reproduction number, infection spikes

## Abstract

Respiratory Syncytial Virus (RSV) is an acute respiratory infection that infects millions of children and infants worldwide. Recent research has shown promise for the development of a vaccine, with a range of vaccine types now in clinical trials or preclinical development. We extend an existing mathematical model with seasonal transmission to include vaccination. We model vaccination both as a continuous process, applying the vaccine during pregnancy, and as a discrete one, using impulsive differential equations, applying pulse vaccination. We develop conditions for the stability of the disease-free equilibrium and show that this equilibrium can be destabilised under certain extreme conditions, even with 100% coverage using an (unrealistic) vaccine. Using impulsive differential equations and introducing a new quantity, the *impulsive reproduction number*, we showed that eradication could be acheived with 75% coverage, while 50% coverage resulted in low-level oscillations. A vaccine that targets RSV infection has the potential to significantly reduce the overall prevalence of the disease, but appropriate coverage is critical.

## 1. Introduction

Respiratory Syncytial Virus (RSV) is the main cause of acute lower respiratory infections in infants and toddlers [1], with almost all children having been infected by two years of age [2,3] and an estimated 0.5–2% of infants requiring hospitalisation due to infection [4]. It has been estimated that, in 2005, 33.8 million new episodes of RSV (both severe and non-severe) occurred worldwide in children younger than five years of age [1]. Symptoms of RSV range from mild (cough, runny nose, sore throat, earache and fever) to more severe afflictions such as bronchiolitis, difficulty breathing, blue skin due to lack of oxygen and pneumonia [2]. While mortality due to RSV infection in developed countries is low, occurring in less than 0.1% of cases [5], few data have been published about RSV morbidity and mortality in developing countries [6]. However, estimates of the hospitalisation costs are substantial [7,8,9], making RSV a significant economic and healthcare-system burden.

Newborn infants are typically protected from RSV infection by maternal antibodies until about six weeks of age [10], and the highest number of observed RSV cases occur in children aged six weeks to six months [11,12]. Immunity to RSV following an infection is short-lasting, and reinfection in childhood is common [13]. Few studies have been undertaken to investigate transmission of RSV among adults, but it is thought that infection can occur throughout life [14,15] and that, in older children and adults, RSV manifests as a mild cold [2,16]. RSV has been identified as a cause of mortality in the elderly, with documented outbreaks in aged-care settings [17,18]; one such study found that up to 18% of pneumonia hospitalisation in adults aged above 65 years may be due to RSV infection [19].

In temperate climates, RSV epidemics exhibit distinct and consistent seasonal patterns. Most RSV infections occur during the cooler winter months, whether wet or dry [6], and outbreaks typically last between two and five months [20,21]. In a number of temperate regions, a biennial pattern for RSV cases has been identified [22,23,24]. In tropical climates, RSV is detected throughout the year with less pronounced seasonal peaks, and the onset of RSV is typically associated with the wet season [6,25].

Immunoprophylaxis with the monoclonal antibody Palivizumab, while not preventing the onset of infection, has proven effective in reducing the severity of RSV-related symptoms [26]. However, prophylaxis is expensive and generally only administered to high-risk children, with recommendations varying across jurisdictions. There is currently no licensed vaccine to prevent RSV infection, despite about 50 years of vaccine research. Recent research has focused on the development of particle-based, subunit and vectored vaccines; several such vaccines are being evaluated in clinical trials, with other vaccines in preclinical development [27,28]. Live-attenuated vaccines are also undergoing Phase 1 trials [29]. With the possibility of an RSV vaccine becoming available, mathematical models are powerful tools for assessing the impacts of different vaccine characteristics.

Several compartment models for RSV transmission have been published to date, most using Susceptible-Exposed-Infectious-Recovered (SEIR) dynamics and with a sine or cosine forcing term to account for seasonal variation in transmission [30,31,32,33,34,35,36]. Few studies have used dynamic models to explore vaccination strategies for RSV, and these have generally investigated RSV vaccination from a cost-effectiveness perspective [37,38], for example in the context of a newborn vaccination strategy in the Spanish region of Valencia [39,40]. More recent studies conducted for the settings of rural Kenya and the United States have focussed on the likely benefits of vaccination for particular target groups [41,42,43]. To the best of our knowledge, there are no theoretical models that examine the impact of an RSV vaccine analytically.

Here, we examine the effects of a vaccine on the transmission of RSV in a single age class. We consider several vaccination scenarios, including differing levels of coverage, seasonal oscillations in the transmission rate and a waning of the vaccine. We also compare continuous vaccination to impulsive vaccination in order to determine conditions on the vaccination strength and duration that will control the virus.

## 2. The Nonimpulsive Model

We first extend the SEIRS compartmental model for a single age cohort described by Weber et al. [30] to include a vaccine strategy for RSV where a fixed proportion of individuals entering the model are temporarily immune to infection. This reflects the situation where pregnant women are vaccinated in their third trimester, generating protective maternal antibodies that are transferred transplacentally to the unborn infant, conferring protection from RSV infection in the first few months of life. We assume that the leaving rate μ is unchanged across all classes and that there is no disease-specific death rate. We scale the entry and leaving rates so that the population is constant.

Let *S* represent susceptible, *I* represent infected and *R* represent recovered individuals, with *V*, $I_{V}$ and $R_{V}$ the corresponding compartments for vaccinated individuals. The birth rate is μ, with a proportion *p* vaccinated, of whom ϵ successfully mount an immune response; the death rate is equal to the birth rate. The time-dependent transmissibility function is β(t), with recovery ν and loss of immunity γ. The transmissibility of infected vaccinated individuals is described by $\beta_{V}\left(t\right)$, and the recovery and loss of immunity rates for vaccinated individuals are $\nu_{V}$ and $\gamma_{V}$ respectively. Finally, the waning of the vaccine protectiveness is given by ω. Note that, although the definition of vaccine duration is not fully elucidated for RSV, mathematically it is well-defined as the period spent in the vaccinated classes before returning to the associated unvaccinated classes. This definition is based on an exponentially distributed time spent in the vaccination classes, and hence the duration corresponds to $\frac{1}{\omega }$ years.

**Figure 1:**
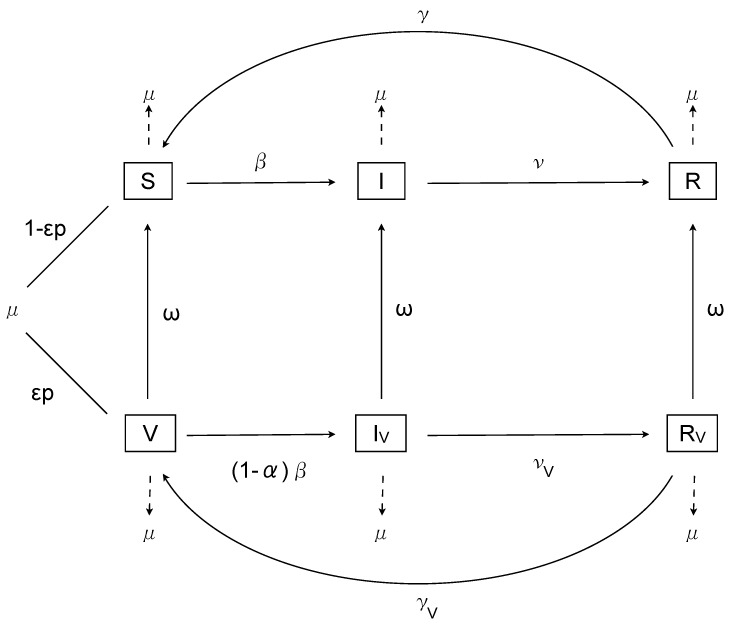
The model.

The basic model with vaccination is then

$$\begin{array}{cc} S^{\prime } & =\mu \left(1-\epsilon p\right)-\mu S-\beta \left(t\right)S\left(I+I_{V}\right)+\gamma R+\omega V\\ I^{\prime } & =\beta \left(t\right)S\left(I+I_{V}\right)-\nu I-\mu I+\omega I_{V}\\ R^{\prime } & =\nu I-\mu R-\gamma R+\omega R_{V}\\ V^{\prime } & =\epsilon p\mu -\mu V-\beta_{V}\left(t\right)V\left(I+I_{V}\right)+\gamma_{V}R_{V}-\omega V\\ I_{V}^{\prime } & =\beta_{V}\left(t\right)V\left(I+I_{V}\right)-\nu_{V}I_{V}-\mu I_{V}-\omega I_{V}\\ R_{V}^{\prime } & =\nu_{V}I_{V}-\mu R_{V}-\gamma_{V}R_{V}-\omega R_{V}, \end{array}$$

with $\beta \left(t\right)=b_{0}\left(1+b_{1}\cos\left(2\pi t+\phi \right)\right)$ and $\beta_{V}\left(t\right)=\left(1-\alpha \right)\beta \left(t\right)$, for 0≤α≤1, where α represents the efficacy of vaccination in preventing infection. (We will relax the lower bound on α later in order to examine some theoretical scenarios.) The model is illustrated in Figure 1.

## 3. Analysis

There is a disease-free equilibrium (DFE) that satisfies

$$\begin{array}{c} \left(\bar{S},\bar{I},\bar{R},\bar{V},{\bar{I}}_{V},{\bar{R}}_{V}\right)=\left(\frac{(1-\epsilon p)\mu +\omega }{\mu +\omega },0,0,\frac{\epsilon p\mu }{\mu +\omega },0,0\right). \end{array}$$

### Constant Transmission

If we assume transmission is constant, so that β and $\beta_{V}$ are independent of time, then the Jacobian is $J=[J_{1}|J_{2}]$, where

$$\begin{array}{cc} J_{1} & =\left[\begin{array}{ccc} -\mu -\beta (I+I_{V}) & -\beta \bar{S} & \gamma \\ \beta (I+I_{V}) & \beta \bar{S}-\mu -\nu & 0\\ 0 & \nu & -\mu -\gamma \\ 0 & -\beta_{V}\bar{V} & 0\\ 0 & \beta_{V}\bar{V} & 0\\ 0 & 0 & 0 \end{array}\right]\\ J_{2} & =\left[\begin{array}{ccc} \omega & -\beta \bar{S} & 0\\ 0 & \beta \bar{S}+\omega & 0\\ 0 & 0 & \omega \\ -\mu -\beta_{V}\left(I+I_{V}\right)-\omega & -\beta_{V}\bar{V} & \gamma_{V}\\ \beta_{V}\left(I+I_{V}\right) & \beta_{V}\bar{V}-\nu_{V}-\mu -\omega & 0\\ 0 & \nu_{V} & -\mu -\gamma_{V}-\omega \end{array}\right]. \end{array}$$

At the DFE, we have

$$\begin{array}{cc} J{|}_{DFE} & =\left[\begin{array}{cccccc} -\mu & -\beta \bar{S} & \gamma & \omega & -\beta \bar{S} & 0\\ 0 & \beta \bar{S}-\mu -\nu & 0 & 0 & \beta \bar{S}+\omega & 0\\ 0 & \nu & -\mu -\gamma & 0 & 0 & \omega \\ 0 & -\beta_{V}\bar{V} & 0 & -\mu -\omega & -\beta_{V}\bar{V} & \gamma_{V}\\ 0 & \beta_{V}\bar{V} & 0 & 0 & \beta_{V}\bar{V}-\nu_{V}-\mu -\omega & 0\\ 0 & 0 & 0 & 0 & \nu_{V} & -\mu -\gamma_{V}-\omega \end{array}\right]. \end{array}$$

The characteristic polynomial satisfies

$$\begin{array}{cc} \det(J-\lambda I) & =\left(-\mu -\lambda \right)\left(-\mu -\gamma -\lambda \right)\left(-\mu -\omega -\lambda \right)\left(-\mu -\gamma_{V}-\omega -\lambda \right)\det M, \end{array}$$

where

$$\begin{array}{cc} M & =\left[\begin{array}{cc} \beta \bar{S}-\mu -\nu -\lambda & \beta \bar{S}+\omega \\ \beta_{V}\bar{V} & \beta_{V}\bar{V}-\nu_{V}-\mu -\omega -\lambda \end{array}\right]. \end{array}$$

The first four eigenvalues are always negative. The nontrivial part of characteristic equation satisfies

$$\begin{array}{cc} \lambda^{2}+b_{1}\lambda +c_{1} & =0, \end{array}$$

where

$$\begin{array}{cc} b_{1} & =-\beta \bar{S}+\mu +\nu -\beta_{V}\bar{V}+\nu_{V}+\mu +\omega \\ c_{1} & =\left(\beta \bar{S}-\mu -\nu \right)\left(\beta_{V}\bar{V}-\nu_{V}-\mu -\omega \right)-\beta_{V}\bar{V}\left(\beta \bar{S}+\omega \right)\\ & =\beta \bar{S}\left(-\nu_{V}-\mu -\omega \right)-\left(\mu +\nu \right)\left(\beta_{V}\bar{V}-\nu_{V}-\mu -\omega \right)-\beta_{V}\bar{V}\omega . \end{array}$$

We use the method of the constant term of the characteristic polynomial to determine the reproduction number [44]. Rearranging $c_{1}=0$, we find

$$\begin{array}{cc} R_{0} & =\frac{\beta \bar{S}\left(\nu_{V}+\mu +\omega \right)+\beta_{V}\bar{V}\left(\mu +\nu +\omega \right)}{\left(\mu +\nu \right)\left(\mu +\nu_{V}+\omega \right)}. \end{array}$$

(This is equivalent to the value found using the next-generation method.)

If $c_{1}=0$ and $b_{1}>0$, then we have a bifurcation with the property that the DFE is stable if $R_{0}<1$ and unstable if $R_{0}>1$.

However, it is possible that when $c_{1}=0$, $b_{1}<0$. In this case, $R_{0}$ is not a threshold, and the disease can persist if $R_{0}<1$.

When $c_{1}=0$, we have

$$\begin{array}{cc} b_{1}{|}_{c_{1}=0} & =\frac{1}{\nu_{V}+\mu +\omega }\left[\beta_{V}\bar{V}\left(\nu -\nu_{V}\right)+{\left(\nu_{V}+\mu +\omega \right)}^{2}\right]. \end{array}$$

Note that if $\nu =\nu_{V}$, then $b_{1}>0$. However, it is plausible that vaccinated individuals infected with RSV will recover faster than unvaccinated individuals. Thus $\nu_{V}>\nu$. This raises the possibility that $b_{1}$ could be negative.

If $\nu_{V}\to \infty$, then this is equivalent to vaccinated individuals recovering instantaneously. In this case,

$$\begin{array}{cc} \lim_{\nu_{V}\to \infty }b_{1} & =\lim_{\nu_{V}\to \infty }\frac{\beta_{V}\bar{V}\left(\nu -\nu_{V}\right)}{\omega +\mu +\nu_{V}}+\omega +\mu +\nu_{V}\\ & =\infty -\beta_{V}\bar{V}>0. \end{array}$$

Hence if we define $f\left(\nu_{V}\right)=\frac{\beta_{V}\bar{V}\left(\nu -\nu_{V}\right)+{\left(\omega +\mu +\nu_{V}\right)}^{2}}{\omega +\mu +\nu_{V}}$, then it is clear that f(0)>0 and f(∞)>0. So we would like to know whether *f* has a turning point $\nu_{V}^{*}$ such that $f(\nu_{V}^{*})<0$.

We have

$$\begin{array}{cc} f^{\prime }\left(\nu_{V}\right) & =\frac{\left(\omega +\mu +\nu_{V}\right)[-\beta_{V}\bar{V}+2\left(\omega +\mu +\nu_{V}\right)]-\left[\beta_{V}\bar{V}\left(\nu -nu_{V}\right)+{\left(\omega +\mu +\nu_{V}\right)}^{2}\right]}{{\left(\omega +\mu +\nu_{V}\right)}^{2}}\\ & =\frac{{\left(\omega +\mu +\nu_{V}\right)}^{2}-\beta_{V}\bar{V}\left[\omega +\mu +\nu \right]}{{\left(\omega +\mu +\nu_{V}\right)}^{2}}. \end{array}$$

**Figure 2:**
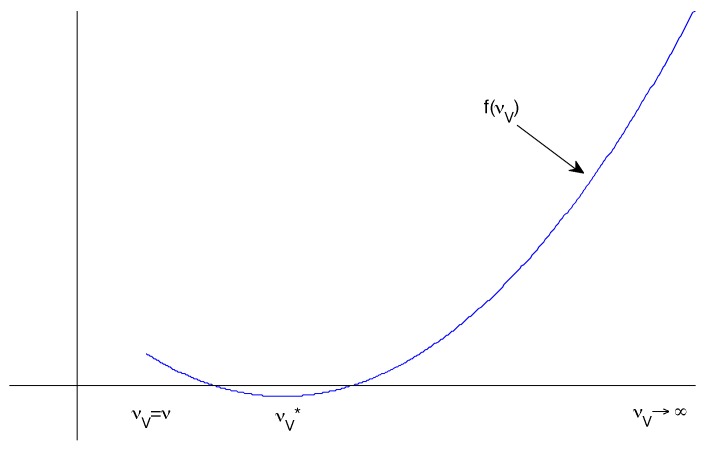
Possible sketch of the form of $f(\nu_{V})$ with a negative minimum between two positive extremes.

It follows that $\nu_{V}^{*}=\sqrt{\beta_{V}\bar{V}\left(\omega +\mu +\nu \right)}-\omega -\mu$. There are three requirements for this to be meaningful (Figure 2):

1. $\nu_{V}^{*}>\nu$
2. $f(\nu_{V}^{*})<0$ and
3. $\nu_{V}^{*}$ is a local minimum.

The first and second criteria determine whether such a $\nu_{V}^{*}$ exists. To prove the third, we can differentiate again:

$$\begin{array}{cc} f^{\prime \prime }\left(\nu_{V}\right) & =\frac{{\left(\omega +\mu +\nu_{V}\right)}^{2}+\beta_{V}\left(\omega +\mu +\nu \right)}{\left(\omega +\mu +\nu_{V}\right)}>0. \end{array}$$

It follows that $\nu_{V}^{*}$ is a local minimum whenever it exists.

## 4. The Impulsive Model

Previously, we assumed that a fixed proportion of pregnant women were vaccinated in pregnancy, resulting in a proportion of infants being born with temporary immunity to RSV infection. This is effectively continuous vaccination. However, vaccination may occur later and may be administered at regular times (for example, in schools or daycare centres). We assume that the effect of the vaccine is to reduce the susceptible population by a fixed proportion *r*. Such a model is described by a system of non-autonomous impulsive differential equations [45,46,47,48].

The impulsive model is given by

$$\begin{array}{cccc} S^{\prime } & =\mu -\mu S-\beta \left(t\right)S\left(I+I_{V}\right)+\gamma R+\omega V & t & \neq t_{k}\\ I^{\prime } & =\beta \left(t\right)S\left(I+I_{V}\right)-\nu I-\mu I+\omega I_{V} & t & \neq t_{k}\\ R^{\prime } & =\nu I-\mu R-\gamma R+\omega R_{V} & t & \neq t_{k}\\ V^{\prime } & =-\mu V-\beta_{V}\left(t\right)V\left(I+I_{V}\right)+\gamma_{V}R_{V}-\omega V & t & \neq t_{k}\\ I_{V}^{\prime } & =\beta_{V}V\left(I+I_{V}\right)-\nu_{V}I_{V}-\mu I_{V}-\omega I_{V} & t & \neq t_{k}\\ R_{V}^{\prime } & =\nu_{V}I_{V}-\mu R_{V}-\gamma_{V}R_{V}-\omega R_{V} & t & \neq t_{k}\\ \Delta S & =-rS & t & =t_{k}\\ \Delta V & =rS & t & =t_{k}. \end{array}$$

Here $t_{k}$ are the vaccination times. They may be fixed or non-fixed, although for our purposes we will consider them fixed.

### 4.1. Impulsive Analysis

We set β to be constant for mathematical convenience, and therefore consider the system in the absence of seasonal transmission. In order to analyse the impulsive system, we need to solve the differential equations for finite time. Since this is not possible in general, we develop several overestimates in order to determine bounds for the long-term numbers of susceptible, infected and vaccinated individuals, under appropriate assumptions.

### 4.2. Susceptible Individuals

First we consider the overestimate $I+I_{V}\leq 1$ (i.e., maximal infection). Then we have

$$\begin{array}{cc} S^{\prime } & \geq \mu -\mu S-\beta S. \end{array}$$

Integrating and applying the "initial" condition $S(t_{k}^{+})$ in the (k+1)st cycle, we have

$$\begin{array}{cc} S(t) & \geq e^{-\left(\mu +\beta \right)\left(t-t_{k}\right)}S\left(t_{k}^{+}\right)+\frac{\mu }{\mu +\beta }\left(1-e^{-\left(\mu +\beta \right)\left(t-t_{k}\right)}\right),\;\mathrm{for}\;t_{k}<t\leq t_{k+1}\\ S(t_{k+1}^{-}) & \geq e^{-(\mu +\beta )\tau }S\left(t_{k}^{+}\right)+\frac{\mu }{\mu +\beta }\left(1-e^{-(\mu +\beta )\tau }\right). \end{array}$$

Applying the impulsive condition, we have

$$\begin{array}{cc} S(t_{k+1}^{+}) & =\left(1-r\right)S\left(t_{k}^{-}\right)\\ S(t_{k+1}^{+}) & \geq \left(1-r\right)e^{-(\mu +\beta )\tau }S\left(t_{k}^{+}\right)+\frac{\mu }{\mu +\beta }\left(1-r\right)\left(1-e^{-(\mu +\beta )\tau }\right). \end{array}$$

A recurrence relation in the form $x_{n+1}=ax_{n}+b$ has equilibrium $\bar{x}=\frac{b}{1-a}$, and the equilibrium is stable if |a|<1. In our case, we have $a=\left(1-r\right)e^{-(\mu +\beta )\tau }<1$, so the equilibrium of the corresponding recurrence relation is stable. It follows that solutions are bounded below by a stable impulsive periodic orbit with endpoints

$$\begin{array}{cc} S_{\infty }^{-} & =\frac{\mu \left(1-e^{-(\mu +\beta )\tau }\right)}{(\mu +\beta )\left(1-\left(1-r\right)e^{-(\mu +\beta )\tau }\right)}\\ S_{\infty }^{+} & =\frac{\mu (1-r)\left(1-e^{-(\mu +\beta )\tau }\right)}{(\mu +\beta )\left(1-\left(1-r\right)e^{-(\mu +\beta )\tau }\right)}. \end{array}$$

These values correspond to the local maximum and minimum values for the unvaccinated susceptibles after a long time. These values are well-defined, since both the numerator and the denominator are always positive.

Note in particular that

$$\begin{array}{c} \lim_{\tau \to 0}S_{\infty }^{-}=0. \end{array}$$

That is, if the period between vaccinations shrinks to zero, then the number of susceptibles would shrink to zero. (Note that this is a theoretical result only, since the impulsive assumptions of long cycle times relative to instantaneous approximation would break down [49].)

### 4.3. Vaccinated Individuals

Second, we consider vaccination. Using the inequalities $I+I_{V}\leq 1$ and $R_{V}\geq 0$, we have

$$\begin{array}{c} V^{\prime }\geq -\mu V-\beta V-\omega V. \end{array}$$

Integrating and applying the "initial" condition $V(t_{k}^{+})$ in the (k+1)st cycle, we have

$$\begin{array}{cc} V(t) & \geq V\left(t_{k}^{+}\right)e^{-\left(\mu +\beta +\omega \right)\left(t-t_{k}\right)},\;\mathrm{for}\;t_{k}<t\leq t_{k+1}\\ V(t_{k+1}^{-}) & \geq V\left(t_{k}^{+}\right)e^{-(\mu +\beta +\omega )\tau }. \end{array}$$

Applying the impulsive condition, we have

$$\begin{array}{cc} V(t_{k+1}^{+}) & =V\left(t_{k+1}^{-}\right)+rS\left(t_{k+1}^{1}\right)\\ V(t_{k+1}^{+}) & \geq V\left(t_{k+1}^{-}\right)+\frac{r\mu \left(1-e^{-(\mu +\beta )\tau }\right)}{(\mu +\beta )\left(1-\left(1-r\right)e^{-(\mu +\beta )\tau }\right)}\\ & \geq V\left(t_{k}^{-}\right)e^{-(\mu +\beta +\omega )\tau }+\frac{r\mu \left(1-e^{-(\mu +\beta )\tau }\right)}{(\mu +\beta )\left(1-\left(1-r\right)e^{-(\mu +\beta )\tau }\right)}. \end{array}$$

Since $e^{-(\mu +\beta +\omega )\tau }<1$, the corresponding recurrence relation has a stable equilibrium, and hence solutions are bounded below by the impulsive periodic orbit with endpoints

$$\begin{array}{cc} V_{\infty }^{-} & =\frac{r\mu \left(1-e^{-(\mu +\beta )\tau }\right)e^{-(\mu +\beta +\omega )\tau }}{(\mu +\beta )\left(1-\left(1-r\right)e^{-(\mu +\beta )\tau }\right)\left(1-e^{-(\mu +\beta +\omega )\tau }\right)}\\ V_{\infty }^{+} & =\frac{r\mu \left(1-e^{-(\mu +\beta )\tau }\right)}{(\mu +\beta )\left(1-\left(1-r\right)e^{-(\mu +\beta )\tau }\right)\left(1-e^{-(\mu +\beta +\omega )\tau }\right)}. \end{array}$$

### 4.4. Infected Individuals

Finally, we examine the number of infected individuals under the assumption that the number of infected vaccinated individuals is negligible (so $I_{V}\approx 0$). We then have

$$\begin{array}{cc} I^{\prime } & \approx \beta SI-\nu I-\mu I\\ & \leq \beta S_{\infty }^{-}I-\nu I-\mu I\\ & =\frac{\beta \mu \left(1-e^{-(\mu +\beta )\tau }\right)}{(\mu +\beta )\left(1-\left(1-r\right)e^{-(\mu +\beta )\tau }\right)}I-\nu I-\mu I. \end{array}$$

It follows that, after sufficient time, the number of infections will be decreasing if

$$\begin{array}{cc} q & =\frac{\beta \mu \left(1-e^{-(\mu +\beta )\tau }\right)}{(\mu +\beta )\left(1-\left(1-r\right)e^{-(\mu +\beta )\tau }\right)}-\nu -\mu <0. \end{array}$$

We thus define a new quantity, the *impulsive reproduction number*

$$\begin{array}{cc} T_{0} & =\frac{\beta \mu \left(1-e^{-(\mu +\beta )\tau }\right)}{(\nu +\mu )(\mu +\beta )\left(1-\left(1-r\right)e^{-(\mu +\beta )\tau }\right)}, \end{array}$$

which has the condition that the disease will be controlled if $T_{0}<1$.

Solving the equation $T_{0}=1$, we can define the maximal period as

$$\begin{array}{c} \hat{\tau }=\frac{1}{\mu +\beta }\ln\frac{(1-r)(\nu +\mu )(\mu +\beta )-\beta \mu }{(\nu +\mu )(\mu +\beta )-\beta \mu }. \end{array}$$

This is defined only if

(1) $$\begin{array}{c} r<r^{*}\equiv 1-\frac{\beta \mu }{\left(\nu +\mu )(\mu +\beta \right)}. \end{array}$$

Differentiating, we have

$$\begin{array}{cc} \frac{\partial T_{0}}{\partial r} & =\frac{\beta \mu \left(1-e^{-\mu +\beta \tau }\right)}{\left(\nu +\mu )(\mu +\beta \right)}\left[-\left(1-\left(1-r\right)e^{-(\mu +\beta )\tau }\right)e^{-(\mu +\beta )\tau }\right]<0. \end{array}$$

It follows that $T_{0}$ is decreasing as *r* increases, for $r<r^{*}$.

Now let $r=r^{*}+\epsilon$ in order to determine what happens beyond $r^{*}$. We have

$$\begin{array}{c} r=\frac{(\nu +\mu )(\mu +\beta )-\beta \mu }{\left(\nu +\mu )(\mu +\beta \right)}+\epsilon . \end{array}$$

Substituting into *q* and taking a common denominator, we find that the numerator of *q* is

$$\begin{array}{c} {\left(\nu +\mu \right)}^{2}{\left(\mu +\beta \right)}^{2}\left[\left(1-\epsilon \right)e^{-(\mu +\beta )\tau }-1\right]<0. \end{array}$$

It follows that $T_{0}<1$ whenever $r>r^{*}$.

In summary, assuming the number of infected vaccinated individuals is negligible, if $r>r^{*}$, where $r^{*}$ is defined by (1), then the disease will be controlled, whereas if $r<r^{*}$, then the disease will theoretically be controlled, assuming the period between vaccinations satisfies $\tau <\hat{\tau }$. High coverage can thus control the disease, while sufficiently frequent vaccinations can achieve control when coverage is limited.

## 5. Numerical Simulations

### 5.1. The Nonimpulsive Model

**Figure 3:**
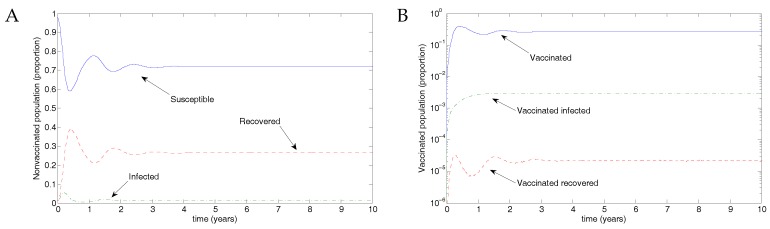
Results from the nonimpulsive model. (**A**) There is an outbreak, and the infectious population oscillates, eventually approaching an endemic equilibrium. (**B**) A small proportion of individuals are (and remain) vaccinated, with a low-level outbreak among vaccinated individuals. Note the log scale in this figure.

From Weber et al. [30], we use the parameter values β=50, μ=1/70 and ν=36, taking the transmission parameter to be constant. Figure 3 shows the results of transmission using disease parameters from Weber et al. [30] and assuming vaccination parameters such that recovery was slightly faster and transmission slightly less likely. Of the eligible population, 50% were assumed to be protected by vaccination, but the vaccine waned after 0.5 years, in line with the natural immunity following recovery from RSV infection. The parameter values were μ=1/70;ω=2;β=50; $\beta_{V}=0.5\beta ;\epsilon =0.9;p=0.5;\nu =36;\nu_{V}=1.2\nu ;\gamma =1.8$; and $\gamma_{V}=1.2\gamma$.

### 5.2. The Impulsive Model

Next, following Weber et al. [30], we examined the more realistic case when the transmission rate oscillated and examined several possibilities for periodic vaccine coverage via the impulse proportion *r*.

**Figure 4:**
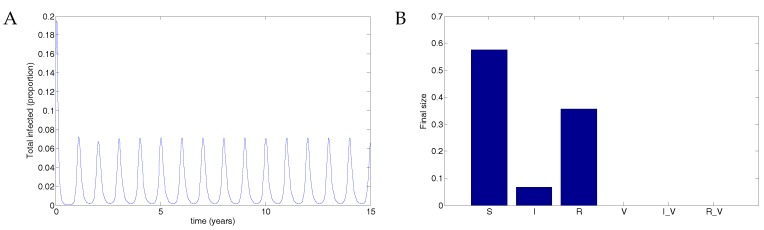
Without vaccination, the disease infects up to 7% of the population. (**A**) The total infected population, including vaccinated individuals; (**B**) The final size in each population.

When there is no vaccine, the disease results in a maximum of about 7% of the population infected. The parameters used were μ=1/70; ω=2; $b_{0}=60;$ $b_{1}=0.16;$ ϕ=0.15; $\beta_{V}=0.5\beta ;\nu =36;$ $\nu_{V}=1.2\nu ;\gamma =1.8;\gamma_{V}=1.2\gamma$ and r=0. See Figure 4.

**Figure 5:**
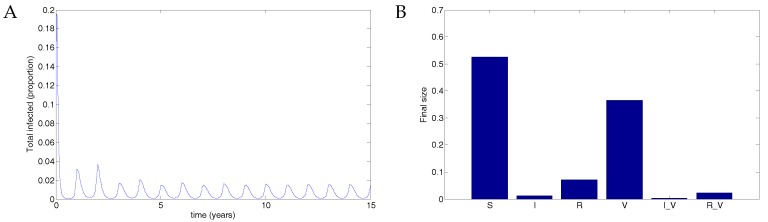
50% coverage with a vaccine that reduced transmissibility by half and waned after two years resulted in a substantial reduction in the disease compared to no vaccination. (**A**) The total infected population, including vaccinated individuals; (**B**) The final size in each population.

A vaccine administered to half the population with 50% transmission that waned after two years resulted in a maximum of about 2% of the population infected. See Figure 5. Data used were identical to Figure 4 except that r=0.5. In this case, the disease still oscillates but at substantially reduced levels.

**Figure 6:**
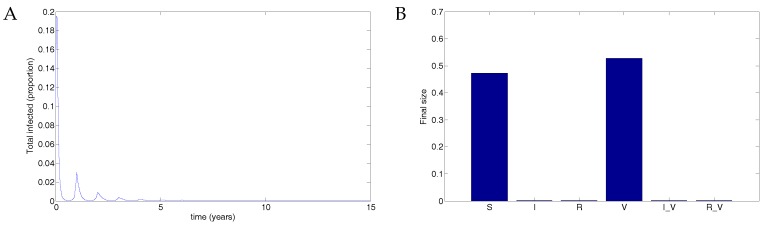
75% coverage with a vaccine that reduced transmissibility by half and waned after two years resulted in theoretical eradication of the disease. (**A**) The total infected population, including vaccinated individuals; (**B**) The final size in each population.

A vaccine given to three quarters of the population with 50% transmission that waned after two years resulted in theoretical eradication of the disease. See Figure 6. The parameters used were identical to those in Figure 4 and Figure 5 except that r=0.75. In this case, there are eventually roughly equal numbers of susceptible and vaccinated individuals, with no infected individuals.

Note that, even in the unrealistic case of perfect coverage with a lifelong vaccine (so that ϵ=p=1 and $\mu =\omega =\frac{1}{70}$), the DFE still satisfies

$$\begin{array}{cc} \bar{S} & =\frac{\omega }{\mu +\omega }=\frac{1}{2}\\ \bar{V} & =\frac{\mu }{\mu +\omega }=\frac{1}{2}, \end{array}$$

so the population without infection would eventually split into equal numbers of vaccinated and unvaccinated susceptible individuals. With infection included and oscillating transmission, explicitly calculating the final size in each compartment is not possible. However, we expect that high coverage with a vaccine with faster waning would tend to a final size with approximately similar numbers; Figure 6 shows that this is indeed the case. Note that these results confirm the theoretical predictions from Section 4.4.

**Figure 7:**
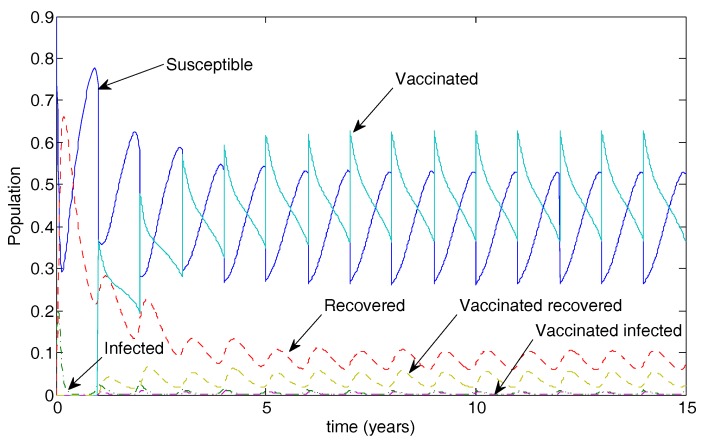
Population dynamics for 50% vaccination coverage for a vaccine that reduced transmissibility by half and waned after two years. Note the low-level oscillations in both infected classes.

Figure 7 illustrates the long-term population dynamics for the case of 50% vaccination coverage. The disease is not eradicated in this case but oscillates at low levels.

**Figure 8:**
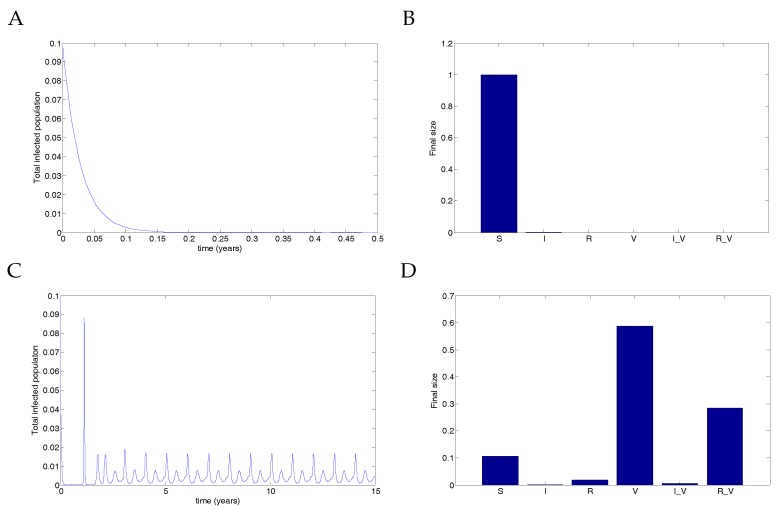
Extreme parameters show that perfect vaccination can induce unexpected infection spikes. (**A**) With no vaccine (r=0), the result is that the infection clears and the entire population remains susceptible. (Note that the timescale is given for only 0.5 years to show the decline but was run for 15 years). (**B**) The final size of each compartment in the case of no vaccine after 15 years. (**C**) When an imperfect vaccine is given to the entire population (r=1), the result is a series of disease spikes in the vaccinated population. Note that the transmission rate is not oscillating in this example. (**D**) The final size of each compartment in the case of full vaccination after 15 years. Vaccination thus destabilises the DFE.

Finally, Figure 8 illustrates the destabilisation of the DFE when extreme vaccination parameters are used. In this case, transmission due to vaccinated individuals was extremely high, but recovery was fast, allowing for low-level infection spikes to occur in the infected populations. The parameters used were $\mu =1/70;\omega =0.1;\beta =0.03;\beta_{V}=300;r=\left\{0,1\right\}$ (representing either no coverage or complete coverage); $\nu =36;\nu_{V}=177;\gamma =1.8;$ and $\gamma_{V}=1.2\gamma .$

With no coverage, the infection clears. However, with complete coverage, the infection rebounds from low levels, producing infection spikes in vaccinated individuals. Although the transmission rate is unrealistically high, this nevertheless demonstrates that a stable DFE can be destabilised by a vaccine. Note that this phenomenon is not a backward bifurcation but rather a destabilisation of the equilibrium.

## 6. Discussion

Before a new vaccine is introduced, anticipated benefits and issues must be assessed. Mathematical models can provide information about the population-level effects of a vaccine and therefore assist in the decision-making process. We have highlighted potential issues that may arise with vaccination for RSV. In particular, we determine conditions under which a destabilisation of the DFE is possible. This is not in the form of a backward bifurcation, as is sometimes seen, but rather occurs when the vaccine causes sufficiently fast recovery but transmission from infected vaccinated individuals is extremely high. An infection-free population that is effectively protected against RSV can nevertheless produce disease spikes in the vaccinated population. These regular spikes occur even in the case when the transmission function is not oscillating. Although such a case is unlikely to occur with the highly unrealistic parameters we chose, we have shown proof-of-concept that it is possible and determined conditions on the recovery rate due to vaccination that allow for the possibility.

We considered two forms of vaccination: single vaccination before infection (such as a maternal vaccine) and periodic vaccination. Using impulsive differential equations, we were able to formulate conditions on the period and the strength of vaccination to allow for disease control.

We also defined a new quantity, the impulsive reproduction number $T_{0}$. This is a sufficient (but not necessary) condition, based on an overestimate of the infected population, that ensures eradication if $T_{0}<1$. If $T_{0}<1$, then the infected population is contracting within each impulsive cycle; the result is the eventual eradication of the infection. Note that we assumed constant transmission for this derivation; however, numerical simulations were performed using seasonal oscillations and demonstrated comparative results. In particular, if the strength of periodic vaccination *r* is sufficiently high, then the disease will be controlled, assuming the vaccine is given with sufficient frequency. See Figure 6.

Our model has some limitations, which should be acknowledged. First, we assumed that the time to administer the vaccine was significantly shorter than the time between vaccine administrations in order to justify the impulsive approximation. Such assumptions are reasonable in many cases [50], although they can produce confounding effects in some situations [49]. The extreme parameters that we used to illustrate the vaccination spikes operated under the assumption that the transmission rate for infected vaccinated individuals was significantly higher than the transmission rate without vaccination. Since we extended the model introduced by Weber et al. [30], our model inherited many of the assumptions from that model, such as mass-action transmission, a constant birth rate and that the birth and death rates were matched, resulting in a constant total population.

In our model, we considered RSV transmission dynamics for a single age class, in order to allow for the model to be analytically tractable. Given that we examined the broad population-level impacts in a large population, we considered this a reasonable model simplification. Furthermore, it has been shown that, for a similar compartmental RSV model, including multiple age classes did not change the bifurcation structure of the model [51]. However, different vaccine candidates for RSV are being developed for distinct key age groups: infants, young children, pregnant women and the elderly [28].

In addition, our model simulated RSV dynamics for a general population, rather than for a specific country or region, so we did not incorporate RSV-related hospitalisation rates for any one region. However, for public-health organisations to make decisions about the cost-effectiveness of a future RSV vaccine, the anticipated reduction in RSV-related hospitalisations will be a key factor. This means that future models that explore the specific implications of vaccines for target age groups may need to incorporate additional age classes and region-specific RSV-related hospitalisation data. The model we present here may be readily adapted to incorporate additional age classes and local public-health data. With regards to the assumption of maternal vaccination, it should be noted that there is some existing level of maternal antibodies that protect some unknown proportion of infants from RSV in their first few months of life (perhaps up to three months [52]). Some other models have accounted for this existing protection [41].

A vaccine that targets RSV infection has the potential to significantly reduce the overall prevalence of the disease, but appropriate coverage is critical. For vaccines of short duration, a single pre-infection vaccine is unlikely to result in eradication. Long-term periodic vaccination can theoretically control the disease, but coverage needs to be sufficiently high. Furthermore, extreme vaccination parameters have the potential to induce unexpected infected spikes as a result of the vaccine. While this is not likely to occur in practice, the possibility of such a surprising result demonstrates the care that should be taken to understand the potential long-term effects of new vaccines before widespread introduction.
